# Evaluation of dietary lignin on broiler performance, nutrient digestibility, cholesterol and triglycerides concentrations, gut morphometry, and lipid oxidation

**DOI:** 10.1016/j.psj.2024.103518

**Published:** 2024-02-01

**Authors:** Brunna Garcia de Souza Leite, Carlos Alexandre Granghelli, Fabricia de Arruda Roque, Rachel Santos Bueno Carvalho, Mário Henrique Scapin Lopes, Paulo Henrique Pelissari, Mylena Tuckmantel Dias, Cristiane Soares da Silva Araújo, Lúcio Francelino Araújo

**Affiliations:** ⁎ School of Animal Science and Food Engineering, University of Sao Paulo, Pirassununga, Sao Paulo, 13635-900, Brazil; † School of Veterinary Medicine and Animal Science, University of Sao Paulo, Pirassununga, Sao Paulo, 13635-900, Brazil

**Keywords:** Antimicrobial, ileal digestibility, Kraft process, lignocellulose, prebiotic

## Abstract

Two trials were performed in order to evaluate the effects of dietary Kraft lignin inclusion on broiler performance, ileal nutrient digestibility, blood lipid profile, intestinal morphometry, and lipid oxidation of meat. Trial 1 was conducted in order to evaluate performance and ileal digestibility for the period of 1 to 21 d of age, randomly distributing 490 day-old broiler chicks across 5 dietary treatments with 14 replicates containing 7 birds each in metabolic cages, while trial 2 was executed in order to evaluate performance, blood parameters, intestinal morphometry, carcass yield and abdominal fat, and lipid oxidation for the period of 1 to 42 d of age, randomly distributing 900 day-old broiler chicks across 5 dietary treatments with 15 replicates of 12 birds each in floor pens, being each bird in trial 2 challenged with coccidiosis vaccine at 10 d of age. The treatments used in both trials were: positive control (**PC**): basal diet + antimicrobial; negative control (**NC**): Basal diet; NC1: NC + 1% lignin; NC2: NC + 2% lignin; NC3: NC + 3% lignin. For trial 1, it was observed that birds fed diets containing 1% lignin had a significant positive effect for BW, feed intake (**FI**), average daily weight gain (**BWG**) and feed conversion rate (**FCR**), similar to the PC, but also showing better EE, CP and AAs ileal digestibility percentages when compared to other treatments. For trial 2, it was observed that during the period of 21 to 35 d, the inclusion of lignin in the diet provided better results in animal performance, similar to the PC group, but at 42 d, animals fed with dietary lignin showed results lower than animals fed the PC diet (*P* < 0.05). Animals fed with increasing lignin concentrations showed decreasing levels of HDL (*P* < 0.05). As of intestinal morphometry, animals fed with 1% and 3% lignin showed longer intestinal length (*P* < 0.05). At 14 d of age, it was observed that animals fed with lignin showed oxidation levels similar to the control treatments. The inclusion of up to 1% lignin in the diet provides beneficial effects on productive performance and nutrient digestibility, while the inclusion of 2% lignin provided lower cholesterol levels, lower villus/crypt ratio, and better internal organ development, therefore, it can be considered an alternative to performance-enhancing antimicrobials in broiler chicken diets.

## INTRODUCTION

Performance-enhancing antimicrobials had been used in animal production for decades in order to control microbial agents that are harmful to the digestive process, and thus allow better use of nutrients from the diet (Jordao-Filho et al., 2017), besides being responsible for providing great benefits to broiler production, expressed mainly as in higher growth rate and feed efficiency, decreased mortality rates and increased productivity (Lorençon et al., 2007; Broom, 2017). Although there are so many positive responses regarding the use of antimicrobials, many questions have been raised due to the possibility of the development of multiresistant microorganisms and the demand for residue-free products by the consumer market (Silva et al., 2003; Ramos et al., 2011). Thus, studies have been conducted in order to find viable alternative sources that, in addition to ensuring food safety, can also optimize the use of nutrients in diets and provide better productivity rates and food efficiency (Araujo et al., 2007).

As a possible alternative, products obtained from lignocellulosic materials such as fructooligosaccharides (**FOS**), mannanoligosaccharide (**MOS**) and xyloligosaccharides (**XOS**), which have been developed as prebiotics, that can be obtained from hemicellulose from different sources of biomass and agro-industrial residues (Otieno and Ahring, 2012; Borsatti et al., 2016) can be highlighted. The main action mechanism of prebiotics is to selectively stimulate the growth and/or activity of a limited group of beneficial bacteria in the intestine (Gibson and Roberfroid, 1995; Gibson et al., 2017), resulting in an improvement in luminal conditions (Van Immerseel et al., 2002) and in the anatomical characteristics of the gastrointestinal tract, promoting an increase in the absorption surface of the intestinal mucosa and effects on the immune system and, in some cases, by improving productive performance (Silva and Nörnberg, 2003).

Lignin is one of several natural compounds that have received scientific interest and is considered as a natural additive, due to its prebiotic effect (Baurhoo et al., 2009). It is composed of a group of phenolic polymers, obtained from pulp and paper industry residues (Tejado et al., 2007), being the second most abundant macromolecular substance and main component of the plant cell wall (Anwar et al., 2014; Shahzadi et al., 2014). Several in vitro and in vivo studies have demonstrated antimicrobial properties of the phenolic fragments in lignin, as well as its influence in the regulation of blood lipid metabolism and oxidative stress, since the molecular structure of lignin contains antioxidant groups that can stabilize free radicals present in the organism and also inhibit the oxidation of lipoproteins, therefore allowing for a slower lipid oxidation (Raza et al., 2018; Zheng et al., 2021). There are different chemical treatments during wood pulping processes that produce different types of lignin (Alcell, purified, or Kraft), in which they exert different responses (Baurhoo et al., 2007a), mainly those linked to the morphological structure of the intestine (Nelson et al., 1994; Baurhoo et al., 2007a; Baurhoo et al., 2007b). Lignin from the Kraft process is currently the least studied type among different species, even though 90% of pulp production comes from this process, generating an immense quantity of byproducts and residues such as hydrolysable lignin (Dessbesell et al., 2020). There are almost no published studies regarding its effects on inclusion in the diet of broilers, as well as its possible prebiotic action and its dynamics in the digestive tract. For this reason, the aim of this study is to evaluate the effect of Kraft lignin supplementation on performance, ileal nutrient digestibility, blood lipid profile, intestinal morphometry, and lipid oxidation of meat in broilers.

## MATERIAL AND METHODS

The study was conducted at the Poultry Research Laboratory of the Department of Nutrition and Animal Production of the School of Veterinary Medicine and Animal Husbandry, University of Sao Paulo, Pirassununga, Brazil. All experimental procedures were approved by the Animal Ethics Committee of the School of Animal Husbandry and Food Engineering (CEUA/FZEA, protocol n° 2244051120).

### Trial 1

#### Experimental Design

A total of 490 one-day old Cobb 500 broilers were allocated in an experimental masonry shed with open sides bounded by screens and equipped with fans for temperature control, in stainless aluminum metabolic cages with dimensions of 34 × 100 × 24 cm, equipped with an infrared heating lamp, nipple drinker and through feeder.

The feeding, lighting program, and handling practices were followed according to the lineage manual (Cobb 500, Cobb Vantress, 2019). Feed and water were provided ad libitum. Temperature, humidity, mortality, and withdrawals data were recorded twice a day.

Birds were selected according to BW and after standardization were distributed in a completely randomized design, in 5 treatments with 14 replicates of seven broilers each, totalizing 70 experimental plots. The treatments consisted of positive control (**PC**): basal diet + antimicrobial; negative control (**NC**): Basal diet; NC1: NC + 1% lignin; NC2: NC + 2% lignin; NC3: NC + 3% lignin. Experimental diets were isocaloric and isonutritional, corn and soybean meal based, mash-fed, and formulated to meet the nutritional recommendations proposed by Rostagno et al. (2017) for broilers from 1 to 21 d of age (Table 1).

**Table 1:** Composition of diets for 1-to-21-day old broiler chicken.

	*Treatments*				
*Ingredients*	PC*	NC	NC1	NC2	NC3
	*————————–(%)—————————*				
Corn	49.610	49.610	49.610	49.610	49.610
Soybean meal	37.950	37.950	37.950	37.950	37.950
Soybean oil	5.120	5.120	5.120	5.120	5.120
Dicalcium phosphate	1.870	1.870	1.870	1.870	1.870
Limestone	0.940	0.940	0.940	0.940	0.940
Salt	0.460	0.460	0.460	0.460	0.460
DL-Methionine	0.400	0.400	0.400	0.400	0.400
L-Lysine HCL	0.350	0.350	0.350	0.350	0.350
L-Threonine	0.150	0.150	0.150	0.150	0.150
Vitamin premix^1^	0.100	0.100	0.100	0.100	0.100
Mineral premix^2^	0.050	0.050	0.050	0.050	0.050
Filler^3^	3.000	3.000	2.000	1.000	0.000
Lignin^4^	0.000	0.000	1.000	2.000	3.000
Antimicrobial^5^	0.050	0.000	0.000	0.000	0.000
Total	100.000	100.000	100.000	100.000	100.000
	*Calculated nutrient content*				
Metabolizable energy(kcal/Kg)	3,050	3,050	3,050	3,050	3,050
Crude protein	22.200	22.200	22.200	22.200	22.200
Calcium	0.959	0.959	0.959	0.959	0.959
Available phosphorus	0.460	0.460	0.460	0.460	0.460
Digestible lysine	1.340	1.340	1.340	1.340	1.340
Digestible methione + Cysteine	0.980	0.980	0.980	0.980	0.980
Digestible threonine	0.872	0.872	0.872	0.872	0.872
Digestible valine	0.890	0.890	0.890	0.890	0.890
Digestible arginine, %	1.38	1.38	1.38	1.38	1.38
Digestible histidine, %	0.55	0.55	0.55	0.55	0.55
Digestible leucine, %	1.70	1.70	1.70	1.70	1.70
Digestible isoleucine, %	0.86	0.86	0.86	0.86	0.86
Digestible tryptophan, %	0.25	0.25	0.25	0.25	0.25
Digestible phenylalanine, %	1.08	1.08	1.08	1.08	1.08
Crude Fiber	3.210	3.210	3.210	3.210	3.210
		*Analyzed nutrient content*			
Crude Protein	24.07	20.79	22.55	21.11	23.34
Gross Energy (Kcal/Kg)	4,500	4,416	4,553	4,642	4,710
Crude Fat	7.86	7.92	7.63	7.93	7.19
Crude Fiber	5.09	5.64	4.28	5.22	5.53
ADF	7.89	6.34	5.69	5.14	5.36
NDF	17.71	17.30	9.15	15.85	15.75

1 Provided per kg of diet: folic acid 700.00 mg; pantothenic acid 8.000 mg; biotin 60.00 mg; niacin 30.00 g; vitamin A 8,000,000.00 IU; vitamin B1 3,000,00 mg; vitamin B12 10,000.00 μg; vitamin B2 4,000.00 mg; vitamin B6 2,000.00 mg; vitamin D3 2,000,000.00 IU; vitamin E 15,000.00 IU; vitamin K3 2,000.00 mg.

2 Provided per kg of diet: copper 12,600.00 mg; iron 105.00 g; iodine 2520.00 mg; manganese 126.00 g; zinc 126.00 g

3 Washed sand as inert filler.

4 A 94.60% lignin, phenolic compounds: 1.16% guaiacol and 1.82% siringol, 3% ashes and carbohydrates (Xylose and glucose).

5 Antimicrobial zinc bacitracin, used only in the PC diet.

The lignin used in this study was extracted from *Eucalyptus urograndis* wood as a by-product of Kraft's cellulose production process. The product is composed of 94.60% lignin, and as for the phenolic compounds: 1.16% guaiacol, 1.82% siringol, and the remaining 3% is composed of ashes and carbohydrates (xylose and glucose).

#### Bird Performance

Productive performance data were obtained at 21 d of age (98 birds per treatment), as follows: average body weight (BW - determined by weighing all the animals and then the result divided by the number of animals weighed per cage), average body weight gain (BWG - obtained by difference between the initial and final weight of birds during the experiment), average feed intake (FI - difference between the amount of feed provided at the beginning and the resulting leftovers at the end of the experiment) and feed conversion rate (FCR - determined from the ratio between feed intake and weight gain in the experimental period and corrected for mortality).

#### Ileal Digestibility

The method of ileal digestibility proposed by Sakomura and Rostagno (2016) was used to determine the nutrients' digestibility. As a nondigestible marker, 1 Kg/ton titanium dioxide was added to all experimental diets. The birds were submitted to 3 d of adaptation with the nondigestible marker, starting on the 18th day of the trial.

Feed consumption was stimulated in order to avoid emptying the gastrointestinal tract. At 21 d of age, all birds (98 birds per treatment) were slaughtered by cervical dislocation. Immediately after slaughter, the ileum was exposed through an abdominal incision, and a segment of approximately 15 cm, starting 4 cm from the ileocecal junction, was sectioned. With light manual pressure on the segment, the contents were collected in a plastic container properly identified by treatment/replicates and stored in a freezer at −30°C until processing.

Thawing was carried out at room temperature. Samples were weighed, homogenized and submitted to a drying process in a forced ventilation oven at 55°C for 72 h. Then, samples were ground in a 1 mm sieve, homogenized, and stored in plastic bags for determination of CP, ether extract (**EE**) and total amino acids (**AAs**) levels. CP and EE were determined according to the methodology described by Silva and Queiroz (2006) and by the Association of Official Analytical Chemists (AOAC, 1995). The amino acid analysis was performed by acid hydrolysis and interpretation of the samples by high performance liquid chromatography (**HPLC**), using Shimatzu liquid phase chromatograph – 10ª, with fluorescence detector. The analysis of titanium dioxide concentrations was performed according to the procedure proposed by Myers et al. (2004).

The ileal digestibility percentages of crude protein (**IDCP**), ether extract (**IDEE**) and total amino acids (**IDAAs**) were calculated based on their concentration in the feed and in the ileal content corrected for the concentration of the nondigestible marker. The calculations were performed using the formulas described below:

a) Calculation of the nondigestibility factor (**NDF**):

*NDF = Feed Marker / Excreta Marker*

a) Apparent digestibility percentage of the analyzed nutrient (**AD**):

*ADC (%) = % nutrient in the diet - (% nutrient in the excreta x NDF) / % nutrient in the diet*

### Trial 2

#### Experimental Design

A total of 900 one-day old Cobb 500 broilers were allocated in an experimental masonry shed with open sides bounded by screens and equipped with fans for temperature control, in 100 × 120 cm surface floor pens, equipped with nipple drinker and tubular feeder. The feeding, lighting program and handling practices were followed according to the lineage manual (Cobb 500, Cobb Vantress, 2019). Feed and water were provided ad libitum. Temperature, humidity, mortality, and withdrawals data were recorded twice a day.

Birds were selected according to BW and after standardization were distributed in a completely randomized design, in 5 treatments with 15 replicates of 12 broilers each, totalizing 75 experimental plots. The experimental diets were the same as the ones used in trial 1. Diets were provided as mash-fed in 3 phases: Starter (1–21 d), grower (22–35 d), and final (36–42 d). The lignin used in this trial was the same type as used for trial 1 (Table 1, Table 2, Table 3). Birds were challenged with coccidiosis vaccines using 10 times the recommended dose (0.03 mL x 10) by gavage at 10 d of age (Biococcivet R, Biovet, Vargem Grande Paulista, São Paulo, Brazil), in order to simulate conditions usually observed in commercial farms.

**Table 2:** Composition of diets for 22 to 35 days old broiler chicken.

	*Treatments*				
*Ingredients*	PC*	NC	NC1	NC2	NC3
	*————————-(%)————————-*				
Corn	53.27	53.27	53.27	53.27	53.27
Soybean meal	33.18	33.18	33.18	33.18	33.18
Soybean oil	6.57	6.57	6.57	6.57	6.57
Dicalcium phosphate	1.50	1.50	1.50	1.50	1.50
Limestone	0.85	0.85	0.85	0.85	0.85
Salt	0.43	0.43	0.43	0.43	0.43
DL-Methione	0.38	0.38	0.38	0.38	0.38
L-Lysine HCL	0.37	0.37	0.37	0.37	0.37
L-Threonine	0.16	0.16	0.16	0.16	0.16
Valine	0.14	0.14	0.14	0.14	0.14
Vitamin premix^1^	0.10	0.10	0.10	0.10	0.10
Mineral premix^2^	0.05	0.05	0.05	0.05	0.05
Filler^3^	3.00	3.00	2.00	1.00	0.00
Lignin^4^	0.00	0.00	1.00	2.00	3.00
Antimicrobial^5^	0.05	0.00	0.00	0.00	0.00
Total	100.000	100.000	100.000	100.000	100.000
	*Calculated nutrient content*				
Metabolizable energy (kcal/Kg)	3200	3200	3200	3200	3200
Crude protein	22.62	22.62	22.62	22.62	22.62
Calcium	0.82	0.82	0.82	0.82	0.82
Available phosphorus	0.38	0.38	0.38	0.38	0.38
Digestible lysine	1.23	1.23	1.23	1.23	1.23
Digestible methionine + cysteine	0.91	0.91	0.91	0.91	0.91
Digestible threonine	0.81	0.81	0.81	0.81	0.81
Digestible valine	0.95	0.95	0.95	0.95	0.95
Digestible arginine, %	1.25	1.17	1.17	1.17	1.17
Digestible histidine, %	0.50	0.48	0.48	0.48	0.48
Digestible leucine, %	1.58	1.53	1.53	1.53	1.53
Digestible isoleucine, %	0.78	0.73	0.73	0.73	0.73
Digestible tryptophan, %	0.22	0.21	0.21	0.21	0.21
Digestible phenylalanine, %	0.92	0.88	0.88	0.88	0.88
Crude fiber	3.00	3.00	3.00	3.00	3.00
		*Analyzed nutrient content*			
Crude protein	18.65	16.43	18.98	20.54	19.02
Gross energy (Kcal/Kg)	4,500	4,521	4,715	4,909	4,829
Crude fat	10.22	10.33	10.50	9.00	9.73
Crude fiber	4.47	5.91	4.63	5.67	6.38
ADF	5.38	7.37	5.22	4.85	4.06
NDF	16.94	18.89	17.07	19.82	13.10

1 Provided per kg of diet: folic acid 700.00 mg; pantothenic acid 8.000 mg; biotin 60.00 mg; niacin 30.00 g; vitamin A 8,000,000.00 IU; vitamin B1 3,000,00 mg; vitamin B12 10,000.00 μg; vitamin B2 4,000.00 mg; vitamin B6 2,000.00 mg; vitamin D3 2,000,000.00 IU; vitamin E 15,000.00 IU; vitamin K3 2,000.00 mg.

2 Provided per kg of diet: copper 12,600.00 mg; iron 105.00 g; iodine 2520.00 mg; manganese 126.00 g; zinc 126.00 g.

3 Washed sand as inert filler.

4 94.60% lignin, phenolic compounds: 1.16% guaiacol and 1.82% siringol, 3% ashes and carbohydrates (Xylose and glucose).

5 Antimicrobial zinc bacitracin, used only in the PC diet.

**Table 3:** Composition of diets for 36 to 42 days old broiler chicken.

	*Treatments*				
*Ingredients*	PC*	NC	NC1	NC2	NC3
	*————————(%)———————–*				
Corn	56.42	56.42	56.42	56.42	56.42
Soybean meal	30.54	30.54	30.54	30.54	30.54
Soybean oil	6.59	6.59	6.59	6.59	6.59
Dicalcium phosphate	1.11	1.11	1.11	1.11	1.11
Limestone	0.70	0.70	0.70	0.70	0.70
Salt	0.41	0.41	0.41	0.41	0.41
DL-Methione	0.35	0.35	0.35	0.35	0.35
L-Lysine HCL	0.37	0.37	0.37	0.37	0.37
L-Threonine	0.20	0.20	0.20	0.20	0.20
Valine	0.16	0.16	0.16	0.16	0.16
Vitamin premix^1^	0.10	0.10	0.10	0.10	0.10
Mineral premix^2^	0.05	0.05	0.05	0.05	0.05
Filler^3^	3.00	3.00	2.00	1.00	0.00
Lignin^4^	0.00	0.00	1.00	2.00	3.00
Antimicrobial^5^	0.05	0.00	0.00	0.00	0.00
Total	100.000	100.000	100.000	100.000	100.000
	*Calculated nutrient content*				
Metabolizable energy (kcal/Kg)	3250	3250	3250	3250	3250
Crude Protein	19.54	19.54	19.54	19.54	19.54
Calcium	0.66	0.66	0.66	0.66	0.66
Available phosphorus	0.31	0.31	0.31	0.31	0.31
Digestible Lysine	1.23	1.23	1.23	1.23	1.23
Digestible methionine + cysteine	0.91	0.91	0.91	0.91	0.91
Digestible threonine	1.18	1.18	1.18	1.18	1.18
Digestible valine	0.93	0.93	0.93	0.93	0.93
Digestible arginine, %	1.17	1.17	1.17	1.17	1.17
Digestible histidine, %	0.48	0.48	0.48	0.48	0.48
Digestible leucine, %	1.53	1.53	1.53	1.53	1.53
Digestible isoleucine, %	0.73	0.73	0.73	0.73	0.73
Digestible tryptophan, %	0.21	0.21	0.21	0.21	0.21
Digestible phenylalanine, %	0.88	0.88	0.88	0.88	0.88
Crude fiber	2.91	2.91	2.91	2.91	2.91
	*Analyzed nutrient content*				
Crude protein	18.92	19.25	18.14	18.34	18.21
Gross energy (Kcal/Kg)	4,620	4,632	4,675	4,811	4,997
Crude fat	9.42	9.42	9.23	9.10	10.02
Crude fiber	6.02	5.60	4.84	5.34	6.15
ADF	4.52	4.48	4.40	4.39	6.28
NDF	13.33	12.59	14.21	15.42	18.81

1 Provided per kg of diet: folic acid 700.00 mg; pantothenic acid 8.000 mg; biotin 60.00 mg; niacin 30.00 g; vitamin A 8,000,000.00 IU; vitamin B1 3,000,00 mg; vitamin B12 10,000.00 μg; vitamin B2 4,000.00 mg; vitamin B6 2,000.00 mg; vitamin D3 2,000,000.00 IU; vitamin E 15,000.00 IU; vitamin K3 2,000.00 mg.

2 Provided per kg of diet: copper 12,600.00 mg; iron 105.00 g; iodine 2520.00 mg; manganese 126.00 g; zinc 126.00 g.

3 Washed sand as inert filler.

4 A 94.60% lignin, phenolic compounds: 1.16% guaiacol and 1.82% siringol, 3% ashes and carbohydrates (Xylose and glucose).

5 Antimicrobial zinc bacitracin, used only in the PC diet.

#### Bird Performance

Productive performance data (BW, FI, BWG and FCR) were obtained at 7, 21, 35 and 42 d of age. FCR was corrected for mortality in the respective period. The productive performance index (**PPI**) was also calculated by the following formula:

PPI=((BWG×liveability)/(FCR×daysofage))×100

#### Blood Lipid and Gut Morphology Analysis

At 21 and 42 d of age, 1 bird from each of 8 replicates per treatment (8 birds per treatment) were randomly chosen and blood samples were collected and homogenized in heparinized vacutainer tubes for high density lipoprotein (**HDL**), low density lipoprotein (**LDL**), very low-density lipoprotein (**VLDL**) and triglycerides analysis. HDL, total cholesterol, and triglycerides determinations were performed via a colorimetric-based commercial kit (Labtest, Vargem Grande Paulista, São Paulo, Brasil). VLDL and LDL determinations were performed as described by Friedewald et al. (1972). One bird from each of 8 replicates per treatment (8 birds per treatment) were also randomly chosen, weighted, and euthanized by cervical dislocation for intestinal morphometry analysis: Gizzard weight, intestinal weight and length were recorded and then approximately 2 cm per intestinal fragment (Duodenum, jejunum and ileum) were sectioned for histological analysis of villus height and crypt depth, as proposed as routine techniques by Prophet et al. (1992). For each segment, microphotography was taken using objective 10x lens with high resolution digital camera attached within the microscope (Leica DM500 and Leica ICC50 HD, Leica, Vargem Grande Paulista, São Paulo, Brasil). The height and depth of 10 villus and 10 crypts, respectively, per bird were measured using the ImageJ software (NIH, Vargem Grande Paulista, São Paulo, Brasil).

#### Carcass and Meat Lipid Oxidation Analysis

At 42 d of age, 2 birds per replicate (30 birds per treatment) were chosen, individually weighted, and slaughtered for carcass yield and abdominal fat analysis. At the moment of carcass weighting, 5 birds per treatment were chosen and their breasts were deboned and cut into 3 equal parts for lipidic oxidation analysis. The parts were placed into polystyrene trays and wrapped in cling film for 0, 7, and 14 d at 2°C and 1.000 lux conditions. After each exposure period, samples were homogenized with trichloroacetic acid, filtered, homogenized with thiobarbituric acid, and placed in water bath at 100°C for 40 min for determination of thiobarbituric acid reactive substances, according to the methodology proposed by Vyncke (1975) and modified by Sorensen and Jorgensen (1996). An 8-point standard curve was also made using a tetraethoxypropane solution for determination of malonaldehyde concentration in samples. Results were expressed as µg malonaldehyde/Kg, and absorbance was measured using 532 nm and 600 nm wave lengths.

#### Statistical Analysis

For both trials, statistical analysis of the data was performed using the Statistical Analysis System software (SAS, Version 9.4, Vargem Grande Paulista, São Paulo, Brasil) considering a significance level of 5%. For each variable studied, after verifying the homogeneity of variances using the Levene test and the normality of data residues using the Shapiro-Wilk test, an analysis of variance was performed (**ANOVA**). In case of significant difference (*P* < 0.05), treatment means were compared using Tukey's test, and lignin levels were evaluated using polynomial equations.

## RESULTS AND DISCUSSION

### Trial 1

#### Bird Performance

Bird liveability in this trial was 100%. In the evaluated period, no differences were observed for FI. However, for the variables of BW, BWG and FCR, significant differences were observed between all treatments (Table 4). Dietary supplementation of 1% lignin showed outcomes similar to the PC group, as well as better results among the tested inclusion levels of lignin. A polynomial cubic response for all the variables was observed when comparing inclusion levels, and through tests of the derivations of the equations, the maximum values were found to be 0.65 for BW, 0.66 for BWG, and 0.63% for FCR.

**Table 4:** Influence of lignin inclusion in broilers diets on bird performance from 1 to 21 d of age (Trial 1).

*Variables*	*Treatments*^1^					*SEM*^2^	*P-value*	*CV (%)*^3^
	PC	NC	NC1	NC2	NC3			
FI (g)	1146.5	1105.7	1119.1	1094.4	1104.9	0.05	0.144	4.94
BW (g)^4^	957.2^a^	884.8^bc^	916.1^ab^	854.8^c^	878.7^bc^	0.04	<.001	4.50
BWG (g)^5^	909.5^a^	837.3^bc^	878.2^ab^	807.2^c^	830.8^c^	0.04	<.001	4.93
FCR (g/g)^6^	1.25^c^	1.32^ab^	1.28^bc^	1.35^a^	1.33^ab^	0.04	<.001	3.69
Broilers (n)	98	98	98	98	98			

a,b,c Means followed by different letters in the same line differ by the Tukey test (*P* < 0.05);

1 PC: positive control diet; NC: negative control diet; NC1: negative control diet + 1% lignin; NC2: negative control diet + 2% lignin; NC3: negative control diet + 3% lignin.

2 SEM; standard error of mean.

3 CV: coefficient of variation; FI: feed intake; BW: body weight; BWG: body weight gain; FCR: feed conversion rate.

4 BW: Y = 0.885 + 0.137.x – 0.135.x^2^ + 0. 030.x^3^; R^2^ = 0.240.

5 BWG: Y = 0.837 + 0.166.x – 0.159.x^2^ + 0.034.x^3^; R^2^ = 0.283.

6 FCR: Y = 1.322 – 0.150.x + 0.150.x^2^ – 0. 033.x^3^; R^2^ = 0.185.

Similar to the findings of Baurhoo et al. (2007a), the FI did not differ between treatments. However, the inclusion of 1.25 or 2.5% dietary lignin in the mentioned study showed performance results similar to the positive control group, while in the present trial, the inclusion of 2% lignin showed the worst performance outcome amongst treatments. Numerous factors can interfere with the effectiveness of prebiotics, such as microbiota's adaptation to the added compound, sanitary challenge conditions, excessive inclusion potentially leading to microbiota becoming unbalanced, dosage and administration methods for additives, and differences in the chemical structure and physical-chemical properties of the compounds (Silva and Nörnberg, 2003; Hooge, 2004; Morales-López et al., 2009).

The BW was significantly higher with 1% inclusion in the diet when compared to the other inclusion levels, where the maximum point found in the equation was 0.65%. A similar result was found by Makivic et al. (2018), who included lignocelluloses in the diet of broilers and observed that the maximum inclusion of 0.60% showed higher body weight after 42 d when compared to the control group.

For FCR, it can be observed that the inclusion of 1% of lignin in the diet showed a better response when compared to the other levels, and as the inclusion level increased, FCR became worse. Those performance results do not corroborate with what has been observed by Nunes and coworkers (2022), who supplemented broiler chickens with dietary purified lignin levels of 0.5, 1.0 and 1.5% from 22 to 42 d of age and did not observe a significant difference for BW and FCR. This may suggest that both the bird age and the type of lignin may interfere with animal performance outcomes in broiler nutrition.

#### Ileal Digestibility

The results of the apparent ileal digestibility percentages of EE, CP and AAs are presented in Table 5. Birds fed a diet containing 1% lignin showed higher digestibility percentages than all other groups for EE, CP, and AAs (*P* < 0.05). By means of the tests of the equation's derivatives, the maximum point values were found, being 0.77% for the ileal digestibility percentage of the ether extract, 0.79% for the ileal digestibility of crude protein, and 0.80% for the ileal digestibility of amino acids.

**Table 5:** Influence of lignin inclusion in broilers diets on the apparent ileal digestibility at 21 d of age (Trial 1).

Variables	Treatments^1^					SEM^2^	*P*-value	CV (%)^3^
	PC	NC	NC1	NC2	NC3			
IDEE (%)^4^	71.65^b^	70.44^c^	72.83^a^	70.23^c^	70.29^c^	0.83	<0.001	1.16
IDCP (%)^5^	88.20^b^	86.97^c^	89.52^a^	87.10^c^	87.18^c^	0.74	<0.001	0.84
IDAAs (%)^6^	89.60^b^	88.01^c^	90.93^a^	88.23^c^	88.15^c^	0.78	<0.001	0.88
Broilers (n)	98	98	98	98	98			

a,b,c Means followed by different letters in the same line differ by the Tukey test (*P* < 0.05);

1 PC: positive control diet; NC: negative control diet; NC1: negative control diet + 1% lignin; NC2: negative control diet + 2% lignin; NC3: negative control diet + 3% lignin.

2 SEM: standard error of mean.

3 CV: coefficient of variation; IDEE: ileal digestibility percentage for ether extract; IDCP: ileal digestibility percentage for crude protein; IDAAs: ileal digestibility percentage for amino acids.

4 EE: Y = 70.441 + 7.429.x – 6.315.x^2^ + 1. 274.x^3^; R^2^ = 0.637.

5 CP: Y = 86.974 + 7.519.x – 6.216.x^2^ + 1.244.x^3^; R^2^ = 0.676.

6 AAs: Y= 88.010 + 8.496.x – 6.945.x^2^ + 1.376.x^3^; R^2^ = 0.710.

Regarding the inclusion of fiber in birds’ diets, it is recommended by literature to provide low to moderate levels of insoluble fiber, since at high levels there is an increase in the speed of feed transit, hindering the action of endogenous enzymes, thus reducing the efficiency of digestion and nutrient absorption (Jiménez-Moreno et al., 2009; Mateos et al., 2012), as well as decreasing the productive performance of animals.

Lignin is classified as insoluble fiber and due to its insolubility properties, it remains longer in the gizzard, thus allowing a pass rate that promotes greater contact of the feed with the enzymes. The more time the feed remains retained in the gizzard, associated with the release of pepsins in the luminal tract, there is greater fractionation of the fed, increasing the digestion and utilization of nutrients present in the diet (Van Soest, 1994; Mateos et al., 2012). Thus, authors believe that the better digestibility of nutrients found in this study was responsible for the beneficial effects observed in productive performance.

The ileal digestibility percentage of crude protein was higher with the inclusion of 1% lignin in the diet compared to the other inclusion levels, and the maximum point found in the equation was 0.79%. A similar result was observed by Farran et al. (2017), being the isocaloric diets containing 0.80% lignocelluloses increased the apparent and true digestibility of protein and amino acids when compared to the control diet in roosters. These results may be related to improvements in the development and functionality of the gizzard and intestine. Bogusławska-Tryk et al. (2016) did not observe a difference in ileal fat digestibility when supplementing broilers with 0.25 or 0.5% dietary lignocelluloses but observed an increase when feeding birds with 1.0% lignocelluloses, similar to what was found in the present study.

### Trial 2

#### Bird Performance

Regarding animal performance (Table 6), no effects were observed for any of the analyzed effects at 7 d of age (*P* > 0.05). However, at 21 d, birds from the PC and NC2 showed higher FI and higher FCR when compared to the NC group (*P* < 0.05), being the maximum values found to be 1.89% for FI. At 35 d of age, differences for all variables were observed: For BW and BWG, birds fed with the PC diet showed higher values when compared to birds fed NC and NC+ (1–3%) Lignin diets (*P* < 0.01). For FI, birds fed with the PC, NC+1% lignin, and NC+2% Lignin diets showed higher values when compared to the other groups (*P* < 0.01). Birds fed with the NC diet showed lower FCR when compared to birds fed with other experimental diets (*P* < 0.01). At 42 d of age, birds fed with PC showed higher BW and BWG when compared to birds fed with dietary lignin (*P* < 0.05), and also higher PPI (*P* < 0.01). From 1 to 42 d of age, the maximum value of the regression equation found for FCR was 2.27%. The beneficial effects usually attributed to plant extracted additives are associated with many classes of active principles that can provide antimicrobial, antifungal, anti-inflammatory, and antioxidant effects (Burt, 2004; Toledo et al., 2007). Those substances might act by stimulating pancreatic enzymes, modulating the intestinal microbiota, reducing pathogenic microorganisms, and enhancing immunological response (Jang et al., 2007). However, phenolic compound effects may vary due to dosage, the origin of the active compound, dietary differences, animal species, and many others. It is observed that the phenolic compounds in lignin were not able to enhance broiler performance by at least as high as the values observed by the control groups. This does not mean that the lignin’ phenolic compounds do not have such properties mentioned above, since birds in the present trial have been individually submitted to a coccidiosis challenge, which could be a possible reason for the inefficiency of lignin under such experimental conditions. It was expected by the authors that the PC performed well under the challenge conditions, however such poor performance for the lignin groups was not expected and may suggest that the challenge was too severe for birds to show their maximum potential.

**Table 6:** Influence of dietary lignin inclusion in broilers diets from 1 to 7, 21, 35 and 42 d of age on bird performance (Trial 2).

*Variables*	*Treatments*^1^					*SEM*^2^	*P-value*	*CV (%)*^3^
	PC	NC	NC1	NC2	NC3			
*1–7 d*								
FI (g)	148.8	146.7	150.4	149.0	151.3	0.011	0.719	7.26
BW (g)	190.0	187.2	189.7	189.4	187.9	0.007	0.670	3.81
BWG (g)	148.8	145.6	148.1	147.9	146.3	0.007	0.603	4.87
FCR (g/g)	1.003	1.007	1.017	1.007	1.035	0.067	0.576	6.65
*1–21 d*								
FI (g)^4^	1,273.7^a^	1,222.6^b^	1,259.3^ab^	1,273.2^a^	1,254.7^ab^	0.057	0.036	4.53
BW (g)	1,096.3	1,070.0	1,081.9	1,094.6	1,066.0	0.041	0.072	3.81
BWG (g)	1,059.2	1,028.5	1,042.4	1,053.9	1,025.7	0.043	0.057	4.14
FCR (g/g)^5^	1.203^ab^	1.181^b^	1.200^ab^	1.208^a^	1.218^a^	0.028	0.002	2.31
*1–35 d*								
FI (g)	3,362.1^a^	3,173.7^b^	3,212.1^ab^	3,270.8^ab^	3,187.7^b^	0.175	0.005	5.39
BW (g)	2,450.4^a^	2,346.2^b^	2,295.8^b^	2,321.7^b^	2,264.9^b^	0.097	<0.001	4.14
BWG (g)	2,456.0^a^	2,349.2^b^	2,298.6^b^	2,330.0^b^	2,267.4^b^	0.103	<0.001	4.38
FCR (g/g)^6^	1.369^ab^	1.351^b^	1.397^a^	1.404^a^	1.407^a^	0.048	0.001	3.44
*1–42 d*								
FI (g)	4,411.2	4,287.1	4,463.7	4,396.1	4,420.7	0.284	0.373	6.45
BW (g)	3,084.9^a^	2,981.2^ab^	2,934.3^b^	2,959.1^b^	2,909.3^b^	0.119	0.001	4.02
BWG (g)	3,090.7^a^	2,986.5^ab^	2,935.6^b^	2,967.3^b^	2,928.5^b^	0.136	0.001	4.56
FCR (g/g)	1.428^b^	1.452^ab^	1.521^a^	1.482^ab^	1.511^a^	0.082	0.002	5.54
PPI	512.78^a^	484.43^ab^	453.56^b^	472.78^b^	456.65^b^	41.670	<0.001	8.75
Mortality^7^ (%)	0.8	0.4	0.7	0.2	0.2			
Broilers (n)	180	180	180	180	180			

a,b,c Means followed by different letters in the same line differ by the Tukey test (*P* < 0.05);

1 PC: positive control diet; NC: negative control diet; NC1: negative control diet + 1% lignin; NC2: negative control diet + 2% lignin; NC3: negative control diet + 3% lignin.

2 SEM: Standard error of mean.

3 CV: coefficient of variation; FI: feed intake; BW: body weight; BWG: body weight gain; FCR: feed conversion rate; PPI: productive performance index.

4 FI: Y = 1.222 + 0.053.x – 0.014.x^2^; R^2^ = 0.100.

5 FCR: Y = 1.184 + 0.012.x; R^2^ = 0.211.

6 FCR: Y = 1.353 + 0.050.x – 0.011.x^2^; R^2^ = 0.201.

7 Mortality data were not analyzed and are presented as observational data only.

#### Blood Lipid Analysis

Concerning the blood parameters, no differences were observed for any of the analyzed variables at 21 d (Table 7). However, at 42 d, it was observed that birds fed with 3% dietary lignin showed lower concentrations of cholesterol when compared to birds fed with other diets (*P* < 0.05).

**Table 7:** Influence of lignin inclusion in broilers diets on blood parameters at 21 and 42 d of age (Trial 2).

*Variables (mg/dL)*	*Treatments*^1^					*SEM*^2^	*P-value*	*CV (%)*^3^
	PC	NC	NC1	NC2	NC3			
*21 d*								
TRI	80.91	84.03	69.70	59.86	85.07	27.29	0.302	35.94
COL	96.71	90.47	94.28	97.07	96.75	12.39	0.802	13.03
HDL	64.68	59.00	61.30	66.92	64.82	8.70	0.394	13.73
LDL	15.77	14.60	18.98	18.15	14.92	8.00	0.747	48.57
VLDL	16.25	14.71	14.00	12.00	17.00	4.73	0.262	31.95
Broilers (n)	8	8	8	8	8			
*42 d*								
TRI	68.68	93.77	88.37	93.61	67.60	24.35	0.090	29.52
COL^4^	111.91^a^	110.74^a^	98.31^ab^	95.96^ab^	92.12^b^	11.80	0.005	11.56
HDL	72.26^a^	66.06^ab^	62.97^ab^	64.34^ab^	60.07^b^	7.47	0.040	11.44
LDL	25.90	21.80	17.58	12.68	18.77	8.54	0.062	43.73
VLDL	13.75	18.85	17.75	18.75	13.28	4.81	0.068	29.17
Broilers (n)	8	8	8	8	8			

a,b,c Means followed by different letters in the same line differ by the Tukey test (*P* < 0.05);

1 PC: positive control diet; NC: negative control diet; NC1: negative control diet + 1% lignin; NC2: negative control diet + 2% lignin; NC3: negative control diet + 3% lignin.

2 SEM: standard error of mean.

3 CV: coefficient of variation; TRI: triglycerides; COL: cholesterol; HDL: high density lipoproteins; LDL: low density lipoproteins; VLDL: very low-density lipoproteins.

4 COL: Y = 108.055 – 5.882.x; R^2^ = 0.242.

HDL acts by withdrawing excessive cholesterol from the organism, leading to the liver where it can be degraded (Lima and Couto, 2006). In this study, it was expected a higher concentration of HDL values, since it is reported that the phenolic compounds present in lignin can reduce the lipoprotein lipidic peroxidation (Oliveira et al., 1999) and therefore lipoproteins could have a longer lifespan in blood plasma. However, it was observed that the group fed with 3% dietary lignin showed the lowest mean among all treatments for HDL concentrations. This is probably due to the metabolic modulation and release of phenolic compounds that can promote an increase in the activity of lecithin-cholesterol acyltransferase, an enzyme that is present at the surface of HDL molecules (Sudheesh et al., 1997). Therefore, more extrahepatic cholesterol is transported to the liver, being both HDL and cholesterol degraded (Manthei et al., 2018).

#### Gut Morphology Analysis

Regarding the intestinal morphometry and development analysis, at 21 d of age, birds from the PC group showed higher gizzard weight (*P* < 0.05) when compared to other treatments (Table 8). Birds from the NC group showed the lowest intestinal weight between treatments (*P* < 0.05). Birds fed with 1 and 2 % dietary lignin showed higher values of intestinal length when compared to birds fed with control diets (*P* < 0.05). At 42 d of age, birds fed with 1 and 3% of dietary lignin showed higher values of intestinal length (*P* < 0.05). The maximum values found were 2.03 for GW, 2.06% for IW, and 1.90% for IL.

**Table 8:** Influence of lignin inclusion in broilers diets on internal organ and gut development at 21 and 42 d of age (Trial 2).

*Variables*	*Treatments*^1^					*SEM*^2^	*P-value*	*CV (%)*^3^
	PC	NC	NC1	NC2	NC3			
*21 d*								
BW (g)	1211^a^	1095^b^	1205^a^	1201^a^	1198^a^	0.05	0.002	4.92
GW (g)^4^	30.27^a^	25.94^b^	26.54^ab^	27.78^ab^	25.38^b^	2.83	0.012	10.41
IW (g)	115.32^ab^	109.03^b^	124.24^a^	122.15^ab^	123.19^ab^	10.44	0.029	8.79
IL (m)^5^	1.84^b^	1.84^b^	2.04^a^	2.05^a^	2.00^ab^	0.15	0.012	7.99
Broilers (n)	8	8	8	8	8			
*42 d*								
BW (g)	3479	3360	3403	3245	3300	0.18	0.113	5.36
GW (g)	55.89	54.46	56.46	53.48	50.62	7.52	0.561	13.88
IW (g)	206.19	210.15	241.87	220.86	241.30	30.72	0.068	13.71
IL (m)^6^	2.32^b^	2.42^ab^	2.59^a^	2.53^ab^	2.65^a^	0.19	0.013	7.60
Broilers (n)	8	8	8	8	8			

a,b,c Means followed by different letters in the same line differ by the Tukey test (*P* < 0.05).

1 PC: positive control diet; NC: negative control diet; NC1: negative control diet + 1% lignin; NC2: Negative control diet + 2% lignin; NC3: negative control diet + 3% lignin.

2 SEM: standard mean error.

3 CV: coefficient of variation; BW: body weight; GW: gizzard weight; IW: intestinal weight; IL: intestinal length.

4 GW: Y = 1.101 + 0.114.x – 0.028.x^2^; R^2^ = 0.392.

5 IW: Y = 110.051 + 14.663.x – 3.542.x^2^; R^2^ = 0.246.

6 IL: Y = 1.848 + 0.240.x – 0.063.x^2^; R^2^ = 0.231.

Gut weight or length changes due to the inclusion of dietary fiber sources are not totally comprehended. However, it is supposed that the increase in intestinal length may contribute to overall intestinal weight (Uni et al., 2003). This was also observed in the present study, where different lignin inclusion rates in broiler diets showed similar results regarding intestinal weight and length.

The intestinal length may also be considered an indicator of good intestinal mucosa development, reflecting directly at gut health and nutrient absorption, since the longer the intestine, the higher the surface area for nutrients to be absorbed by cells (Gomes et al., 2007).

No differences in villus height were observed in any of the experimental diets for all the 3 intestinal sections at 21 d of age (Table 9). In the duodenal section, it was observed that birds supplemented with 3% dietary lignin showed shallower crypts when compared to the NC group. In the ileum, birds fed with 3% dietary lignin showed the lower villus height to crypt depth ratio (*P* < 0.05), being the maximum value found at 1.19%.

**Table 9:** Influence of lignin inclusion in broilers diet on intestinal morphometry at 21 d of age (Trial 2).

*Variables* (μm)	*Treatments*^1^							*SEM*^2^	*P-value*	*CV (%)*^3^
	PC	NC		NC1		NC2	NC3			
*Duodenum*										
VH	1,154.38		1,222.81		1,224.14	1,201.77	1,126.52	136.53	0.570	11.78
CD^4^	207.20^ab^		236.67^a^		209.72^ab^	208.37^ab^	168.51^b^	42.15	0.049	20.45
VH:CD	5.58		5.61		5.69	5.97	6.72	1.00	0.167	16.99
*Jejunum*										
VH	873.60		904.44		924.19	913.09	994.24	153.31	0.613	16.63
CD	170.71		188.83		170.09	162.87	182.12	32.37	0.519	18.50
VH:CD	5.16		4.92		5.43	5.70	5.51	0.78	0.312	14.59
*Ileum*										
VH	551.59		527.72		561.00	521.31	481.76	80.24	0.333	15.17
CD	122.49		117.70		123.69	108.95	126.50	14.11	0.132	11.77
VH:CD^5^	4.51^ab^		4.47^ab^		4.54^ab^	4.85^a^	3.80^b^	0.57	0.016	13.00
Broilers (n)	8		8		8	8	8			

a,b,c Means followed by different letters in the same line differ by the Tukey test (*P* < 0.05).

1 PC: positive control diet; NC: negative control diet; NC1: negative control diet + 1% lignin; NC2: negative control diet + 2% lignin; NC3: negative control diet + 3% lignin.

2 SEM: standard error of mean.

3 CV: coefficient of variation; VH: villus height; CD: crypt depth; VH:CD: villus height to crypt depth ratio.

4 CD: Y = 2236.6956– 20.5822.x; R^2^ = 0.2193.

5 VH:CD: Y = 4.3971 + 0.6689.x-0.2798. x^2^; R^2^ = 0.2408

Intestinal villus are constantly renewed, having loss of cells at the apex of villus and the renewal made by cells from the crypts. The speed at which this renewal happens determines the size of villus. The higher the villus, the bigger the absorption area, while shallow crypts indicate better gut health status (Furlan et al., 2002; Viola and Vieira, 2007). In normal conditions, the higher the villus height to crypt depth ratio, the better the nutrient absorption and the less energetic losses due to cellular renewal (Arruda et al., 2003; Kuzmuk et al., 2005)

Phenolic compounds can aid the intestinal mucosa health and development in many ways, naming the antioxidant action protecting intestinal cells, the antimicrobial action, modulating the intestinal microbiota, and the reduction of pathogenic microorganisms (Mclean et al., 2005; Lee et al., 2006). Thus, the phenolic compounds originated from the studied lignin may have act as an indirect trophic agent in the broilers’ gut mucosa.

At 42 d of age, differences were observed regarding the duodenum VH:CD, being lower in birds fed with diets containing 1 or 2% dietary lignin (*P* < 0.05) (Table 10), and the ileum crypt depth, being lower in the PC and NC groups (*P* < 0.05), as well as the ileum VH:CD being lower in birds fed with the NC+2% lignin diet.

**Table 10:** Influence of lignin inclusion in broilers diets on intestinal morphometry at 42 d of age (Trial 2).

*Variables* (μm)	*Treatments*^1^					*SEM*^2^	*P-value*	*CV (%)*^3^
	PC	NC	NC1	NC2	NC3			
*Duodenum*								
VH	1,450.28	1,380.32	1,374.24	1,287.46	1,295.68	138.37	0.133	10.19
CD	218.35	219.44	257.27	240.74	223.25	32.65	0.098	14.08
VH:CD^4^	6.87^a^	6.37^ab^	5.38^b^	5.36^b^	5.82^ab^	14.13	0.003	14.13
*Jejunum*								
VH	962.86	911.19	882.91	925.64	889.82	110.26	0.616	12.05
CD	163.02	172.04	165.77	169.28	153.67	25.90	0.667	15.72
VH:CD	6.03	5.33	5.43	5.51	5.81	0.75	0.332	13.35
*Ileum*								
VH	616.93	684.85	684.15	600.25	651.08	77.81	0.123	12.01
CD^5^	104.19^b^	105.38^b^	110.34^ab^	126.47^a^	108.46^ab^	13.80	0.019	12.43
VH:CD^6^	5.92^a^	6.55^a^	6.20^a^	4.82^b^	6.031^a^	0.68	<0.001	11.53
Broilers (n)	8	8	8	8	8			

a,b,c Means followed by different letters in the same line differ by the Tukey test (*P* < 0.05);

1 PC: positive control diet; NC: negative control diet; NC1: negative control diet + 1% lignin; NC2: negative control diet + 2% lignin; NC3: negative control diet + 3% lignin.

2 SEM: standard error of mean.

3 CV: coefficient of variation; VH: villus height; CD: crypt depth; VH:CD: villus height to crypt depth ratio.

4 VH:CD: Y = 6.3526 – 1.2594.x + 0.3634.x^2^; R^2^ = 0.327.

5 CD: Y = 105.3884 – 15.7357.x + 28.2443.x^2^ – 7.5524.x^3^; R^2^ = 0.263.

6 VH:CD: Y = 6.5527 + 1.3668.x – 2.3220.x^2^ + 0.6028.x^3^; R^2^ = 0.4831.

As reported by Baurhoo et al. (2007a), the inclusion of Alcell lignin (1.25%) in the diet of broilers showed higher values for villus height and more caliciform cells. However, at higher inclusion rates of lignin (2.5%) this effect was not observed. Villus are important gut structures, mainly because of their importance for nutrient absorption. In this study, no differences were observed regarding villus height in neither of the 2 evaluated periods.

The findings observed in the ileum in the present study corroborate with the findings by Sarikahn et al. (2010), that studied different inclusion levels of dietary lignocelluloses (0.25, 0.5 and 0.75%) in broilers, reporting higher ileal villus and higher VH:CD. This same finding was also reported by Sozcu (2019) when evaluating up to 2% dietary inclusion of lignocelluloses for broilers. Those results may be related to the priority function of each of the intestinal segments, since villus and crypts in each segment have their own characteristics and differences. Furthermore, in birds, large quantities of membrane carriers are located in the ileum, making it the main amino acid absorption site and hence the different results when compared to the duodenum and jejunum (Furlan et al., 2002).

#### Carcass and Meat Lipid Oxidation Analysis

As for the carcass yield and abdominal fat analysis, it is observed that all studied variables showed significative effects between treatments (Table 11). Regarding the weight of warm carcass, birds fed with 3% dietary lignin showed the lighter carcass among treatments. In the cold carcass analysis and carcass yield, birds fed with dietary lignin showed lower weights when compared to the PC group. Concerning abdominal fat, birds fed with 2 and 3% showed lower abdominal fat values between all treatments.

**Table 11:** Influence of lignin inclusion in broilers diets on carcass yield and abdominal fat at 42 d of age (Trial 2).

*Variables*	*Treatments*^1^					*SEM*^2^	*P-value*	*CV (%)*^3^
	PC	NC	NC1	NC2	NC3			
WCW (Kg)^4^	3.11^a^	3.09^a^	2.99^ab^	3.04^ab^	2.95^b^	0.20	0.002	6.60
CCW (Kg)^5^	2.17^a^	2.13^ab^	2.05^bc^	2.09abc	2.01^c^	0.15	<0.001	7.35
CY (%)	69.85^a^	69.06^ab^	68.49^b^	68.77^b^	68.08^b^	1.75	<0.001	2.54
AF (g)^6^	0.036^a^	0.032^a^	0.032^a^	0.031^b^	0.025^b^	0.01	<0.001	33.55
Broilers (n)	30	30	30	30	30			

a,b,c Means followed by different letters in the same line differ by the Tukey test (*P* < 0.05);

1 PC: positive control diet; NC: negative control diet; NC1: negative control diet + 1% lignin; NC2: negative control diet + 2% lignin; NC3: negative control diet + 3% lignin;

2 SEM: standard error of mean.

3 CV: coefficient of variation; WCW: warm carcass weight; CCW: cold carcass weight; CY: carcass yield; AF: abdominal fat.

4 WCW: Y = 3.096 – 0.281.x + 0.229.x^2^ – 0.051.x^3^; R^2^ = 0.074.

5 CCW: Y = 2.139 – 0.244.x + 0.199.x^2^ – 0.044.x^3^; R^2^ = 0.092.

6 AF: Y = 0.033 – 0.002.x; R^2^ = 0.041.

Concerning the lipid oxidation analysis, no differences were observed between treatments in any of the analyzed periods (Table 12), meaning that the inclusion of dietary lignin for broilers did not interfere with meat lipid oxidation. Lipid oxidation is the second main process in which there is loss of meat quality, only after microbial deterioration, causing odor, taste and pigmentation changes (Gray et al., 1996).

**Table 12:** Lipid oxidation on meat from broilers fed different dietary lignin level (Trial 2).

*Variables* *(days)*^6^	*Treatments*^1^					*SEM*^2^	*P-value*	*CV (%)*^3^
	PC	NC	NC1	NC2	NC3			
D7^4^	0.7376	0.6761	0.7359	0.5802	0.7652	0.40	0.8668	56.74
D14^5^	0.8547	0.7008	1.1014	0.5880	0.7275	0.46	0.2056	58.35
Broilers (n)	5	5	5	5	5			

^a,b,c^Means followed by different letters in the same line differ by the Tukey test (*P* < 0.05);

1 PC: positive control diet; NC: negative control diet; NC1: negative control diet + 1% lignin; NC2: negative control diet + 2% lignin; NC3: negative control diet + 3% lignin.

2 SME: standard mean error.

3 CV: Coefficient of variation.

4 D7: Seventh day of storage.

5 D14: Fourteenth day of storage.

6 For this analysis, data from the first day of storage was considered as a covariate.

## CONCLUSION

The results of this study show that the inclusion of up to 1% lignin in the diet of broilers increases productive performance and nutrient digestibility, being similar to the performance found when supplementing the basal diet with feed antimicrobials. The inclusion of 2% lignin was able to lower cholesterol levels, provide better villus to crypt ratio, provide better development of gut sections, and lower the quantity of abdominal fat in the carcass. Therefore, it can be considered as an alternative to performance-enhancing antimicrobials. As for the best functional levels, according to the regression equations, would be 1.89% for FI and 2.27% for FCR, for birds supplemented from 1 to 42 d of age.
